# Surgicel® Granuloma Mimicking Recurrent Thyroid Tumor After Thyroidectomy: A Case Report and Literature Review

**DOI:** 10.7759/cureus.46587

**Published:** 2023-10-06

**Authors:** Ehab Alameer

**Affiliations:** 1 Department of Surgery, College of Medicine, Jazan University, Jazan, SAU

**Keywords:** thyroid surgery, surgicel® granuloma, recurrent tumor, pseudotumoral lesion, postoperative, oxidized regenerated cellulose

## Abstract

Oxidized regenerated cellulose, commonly known by the brand name Surgicel®, is a hemostatic agent widely used in various surgical procedures. While it is generally considered safe and effective, there have been reports of complications associated with its use, including the formation of pseudotumoral lesions. This article presents a case of a patient who developed a Surgicel® granuloma in the thyroid bed, mimicking a recurrent tumor. Surgicel® is known to cause a chronic inflammatory reaction, leading to foreign body giant cell formation and fibroblastic proliferation. Fine-needle aspiration (FNA) cytology is a valuable diagnostic tool for identifying pseudotumoral lesions caused by oxidized cellulose. The characteristic appearance of oxidized cellulose fragments and the presence of a granulomatous reaction can help distinguish these lesions from tumor recurrence or abscesses. To prevent Surgicel® granuloma, it is recommended to use the minimal amount necessary to achieve hemostasis. It is also important to document its use in the operative report. In cases where a recurrent mass lesion is suspected postoperatively, a comprehensive medical history, imaging studies, and FNA are essential for accurate diagnosis and management. This case report highlights the importance of considering Surgicel®-induced granuloma in the differential diagnosis of recurrent thyroid-bed tumors. A correct diagnosis can help avoid unnecessary aggressive interventions, particularly in cancer patients.

## Introduction

Hemostasis is a crucial aspect of thyroid surgery, and various topical hemostatic agents are commonly employed as supplements to conventional methods of hemostasis (such as suture ligation, clips, electrocautery, and energy devices). These agents have gained widespread acceptance for use in both open and minimally invasive surgeries due to their ease of application [1-3]. In thyroid surgery, a commonly utilized absorbable collagen sheet hemostat is Surgicel®. This product provides a scaffold for clot formation and is thought to be efficacious in managing minor bleeding [4]. Although its effectiveness may be a subject of debate, the use of oxidized cellulose in neck or thyroid surgery is generally considered safe [5,6]. The application of hemostatic patches offers several advantages, including reduced operative time, drain output, and hospital stay after thyroid surgery [5].

However, the utilization of oxidized regenerated cellulose as a hemostatic agent has been linked to complications such as foreign body reactions and granuloma formation [7-9], which can occur months or even years after surgery [10]. The exact mechanism behind the development of these granulomas is not well understood. Several case reports [7-17] have described the presence of oxidized cellulose-induced granulomas in various anatomical locations, including the gastrointestinal tract, ovaries, mediastinum, liver, brain, kidney, and nasal cavity.

In this report, we present a case of a symptomatic neck mass that recurred two years after thyroid lobectomy, which was found to be a Surgicel® granuloma.

## Case presentation

A 42-year-old female patient was referred to the endocrine surgery clinic with a suspicious nodule in the right thyroid lobe. She had previously undergone two non-diagnostic fine-needle aspirations (FNAs). After discussing all options, the patient opted for a right thyroid lobectomy and isthmusectomy. The procedure and recovery were uneventful. The pathological evaluation of the surgical specimen revealed thyroid tissue exhibiting follicular adenoma, which was encapsulated and composed of variable-sized follicles lined by flattened to cuboidal epithelial cells with variable amounts of colloid. There was no evidence of capsular or vascular invasion. The rest of the thyroid tissue contained nodules separated by fibrovascular septa, which showed variable-sized follicles lined by cuboidal to flattened epithelial cells, and contained variable amounts of colloid. No atypia or malignancy was present. 

**Figure 1:**
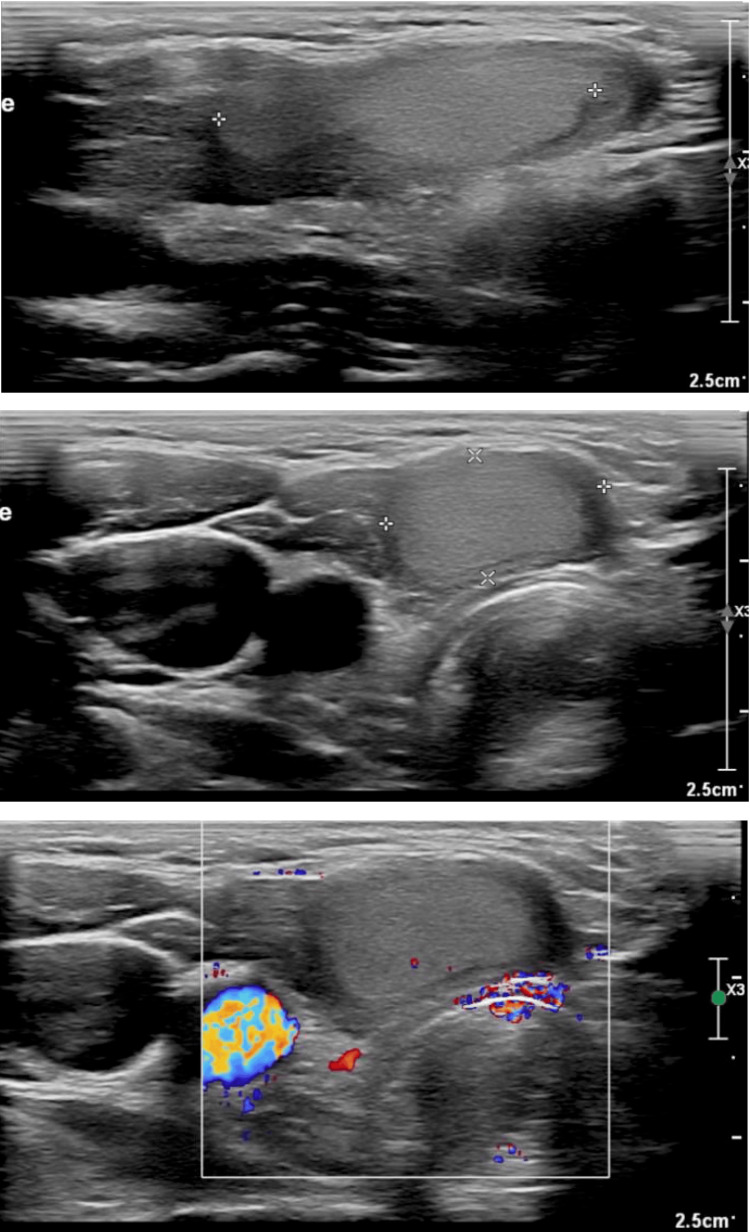
Ultrasound of the pre-tracheal lesion showing an elongated, well-defined isoechoic lesion measuring 2.5 x 1.5 x 0.8 cm in the operative bed The lesion showed no calcifications

Two years after the initial presentation, the patient returned with a visible anterior neck mass underlying the well-healed surgical scar, which was causing “disfigurement and a feeling of pressure in the neck.” Ultrasound (Figure 1) revealed that the right thyroid lobe had been surgically removed, but an elongated, well-defined isoechoic lesion measuring 2.5 × 1.5 × 0.8 cm was present in the operative bed. The lesion showed no calcifications. The left lobe was unremarkable. An FNA of the lesion showed striated muscle fibers, a few macrophages, and degenerated debris, but no epithelial cells were identified.

**Figure 2:**
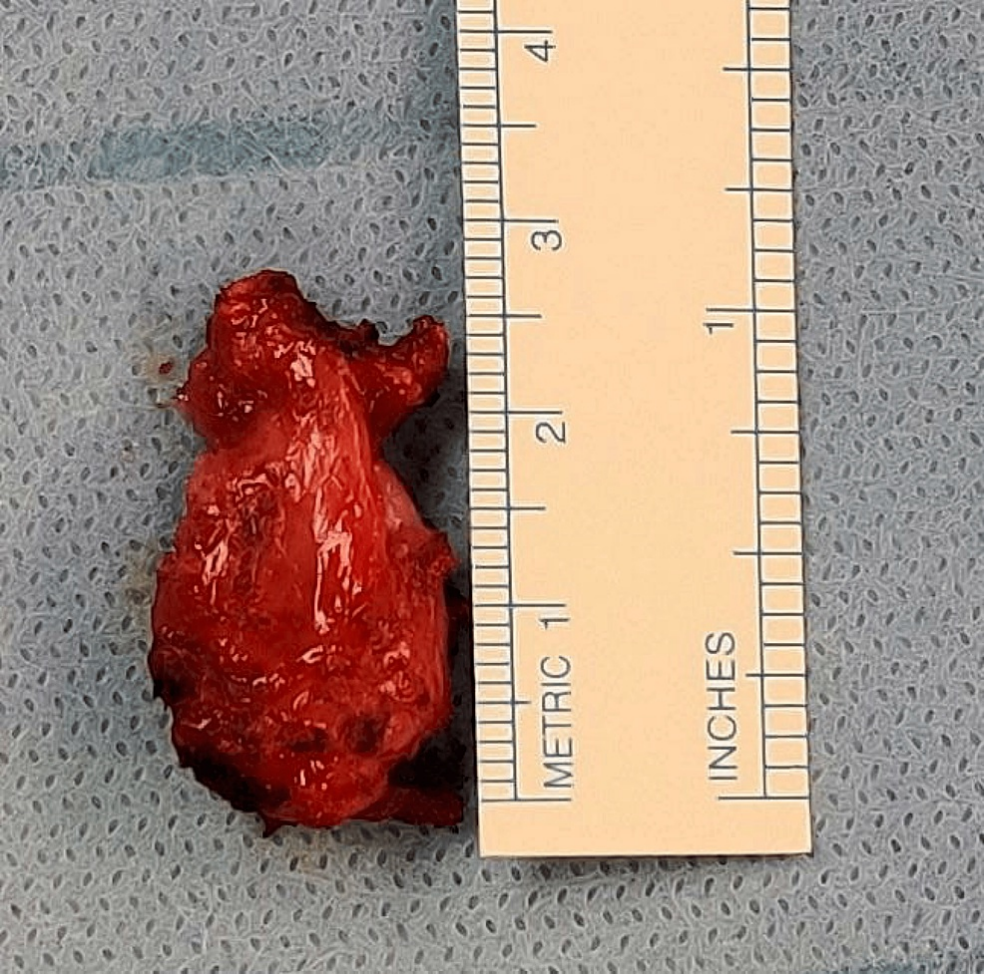
The removed pre-tracheal tumor

After a thorough discussion with the patient, taking into account the symptomatic nature of the mass, the diagnostic uncertainty, and the high level of anxiety, and despite the benign pathology reported following the prior thyroidectomy, we decided to proceed with surgical excision. The tumor was primarily located in the pre-tracheal area, with adhesive extensions into the right tracheoesophageal groove, and was completely removed (Figure 2). The surgery and recovery were unremarkable. The final pathology report revealed chronic xanthogranulomatous inflammation with a foreign body reaction and the presence of foreign material. There were foamy macrophages and a foreign body reaction with multinucleated giant cells surrounding the foreign material, indicative of a granuloma. No atypia or evidence of malignancy was observed, and no lymph node or caseous necrosis was found.

Upon reviewing the operative note from the initial surgery, it was confirmed that Surgicel® was used for hemostasis at the conclusion of the surgery.

## Discussion

The application of hemostatic substances during surgical procedures has become a widespread practice. Among these substances, oxidized cellulose is commonly used due to its biocompatibility, bactericidal properties, and absorption characteristics [18,19]. Surgicel®, a type of oxidized regenerated cellulose, typically dissolves within two to six weeks and has a pH-lowering effect on its surroundings, which may increase inflammation in the surrounding tissue [18]. Surgicel®’s acidic properties cause red blood cells to break down, leading to a change in color when it comes into contact with blood. This change is the result of hemoglobin reacting with acid to form acid hematin [18].

The low pH also exhibits antimicrobial properties and functions as a caustic agent, facilitating blood clotting by creating an artificial clot [18]. However, the acidic environment generated by oxidized cellulose may inactivate other biologically active topical agents, such as thrombin, limiting its ability to be used in combination with other treatments. Furthermore, Surgicel®’s acidic nature may exacerbate inflammation in surrounding tissue and hinder the wound healing process [20].

The duration of oxidized cellulose dissolution depends on the amount used, with a range of two to six weeks. However, there are cases where histological evidence of oxidized cellulose fibers has been observed several years after cardiac surgery [21-23]. Additionally, there have been reports of Surgicel® migrating through the intervertebral foramen and causing spinal cord compression after thoracotomy [24,25].

Besides thyroidectomy, the use of absorbable hemostatic agents, such as Surgicel®, has been investigated in other neck surgeries. For example, one study [26] compared the use of Suturation plus Surgicel® application in managing post-tonsillectomy bleeding and pain. The study found that while the combination of Suturation and Surgicel® reduced the incidence of bleeding, it resulted in increased pain levels compared to other treatment approaches.

Browder and Litwin [27] investigated the effectiveness of Surgicel® in controlling bleeding in general surgical patients, including at the cut surface of the thyroid gland. Their study found that Surgicel® was effective in achieving hemostasis and did not result in any foreign body reactions, infections, or allergic manifestations.

The effectiveness of oxidized regenerated cellulose-based products, such as Surgicel®, in preventing post-surgical adhesion formation has also been investigated in several studies. While some research has indicated that Surgicel® may prevent adhesions, other studies have found inconclusive evidence [28].

In one study, an absorbable collagen-based adhesive was found to reduce adhesions following liver injury in rats [29]. Another study compared the effectiveness of a collagen-based adhesive and conventional absorbable sutures in treating hepatic injury in rats. The adhesive group had shorter hemostasis times and a lower incidence of adhesions [30].

There have been some complications associated with the use of Surgicel® in thyroid surgery. Although studies have demonstrated no significant difference in the incidence of postoperative hypocalcemia when using Surgicel® [6], there have been instances of pseudotumoral lesions induced by oxidized cellulose hemostatic agents.

Notably, Surgicel® granulomas have been documented in various surgical procedures beyond thyroid surgery, including abdominal surgery, neurosurgery, gynecological surgery, and urological surgery [7-17]. These granulomas can exhibit imaging characteristics that resemble recurrent tumors or abscesses, necessitating further examination or resection to differentiate between a foreign-body reaction and tumor recurrence.

In experimental studies using animal models, it has been shown that oxidized cellulose can cause a chronic inflammatory reaction leading to foreign body giant cell formation and fibroblastic proliferation [31].

Hernández-Bonilla et al. [9] described a series of pseudotumoral lesions induced by oxidized cellulose hemostatic agents, including Surgicel®, which can result in a granulomatous reaction. FNA cytology was found to be helpful in detecting these pseudotumoral lesions and differentiating them from tumor recurrence. The authors emphasized the importance of pathologists being familiar with this finding.

The authors evaluated 16 patients who developed these lesions after surgical procedures where oxidized cellulose was used. FNA samples were obtained from various sites, including the mediastinum, thyroid surgical bed, axilla, neck, vulva, liver, and retroperitoneum. The time interval between surgery and FNA ranged from 4 to 46 months. The cytology samples consistently showed foreign-body material with a variable granulomatous reaction. Oxidized cellulose appeared as laminated inorganic fragments with an elongated quadrangular appearance. Additional features included amorphous, ill-defined fragments and a dense proteinaceous background with phagocytic cells.

FNA cytology is a valuable diagnostic tool for identifying pseudotumoral lesions caused by hemostatic agents such as oxidized cellulose. The consistent presence of foreign-body material with a granulomatous reaction and the characteristic appearance of oxidized cellulose fragments can help distinguish these lesions from tumor recurrence or abscesses.

It is essential for physicians to be familiar with the clinical presentation, diagnostic approach, prevention, and management of Surgicel® granuloma. Management often involves excision due to the presence of symptoms or diagnostic uncertainty. To prevent Surgicel® granuloma, it is recommended to use the minimal amount of Surgicel® necessary to achieve hemostasis. Additionally, it is important to note the use of hemostatic agents in the operative report.

This case report adds to the growing body of literature highlighting the importance of considering oxidized cellulose-induced granuloma in the differential diagnosis when evaluating patients with recurrent mass lesions postoperatively.

## Conclusions

In conclusion, Surgicel® granuloma is a possible cause of recurrent thyroid-bed tumors. To accurately diagnose and manage this condition, a comprehensive medical history, imaging studies, and FNA are crucial. This is particularly important in cancer patients, as a correct diagnosis can help avoid unnecessary aggressive interventions.
